# Medicine, morality and health care social media

**DOI:** 10.1186/1741-7015-10-83

**Published:** 2012-08-02

**Authors:** Farris K Timimi

**Affiliations:** 1 Mayo Clinic Center for Social Media, Division of Cardiovascular Diseases and Internal Medicine, Mayo Clinic, 200 First Street, SW, Rochester, MN 55902, USA

**Keywords:** Social media, online, health care providers, patient-physician

## Abstract

Social media includes many different forms of technology including online forums, blogs, microblogs (i.e. Twitter), wikipedias, video blogs, social networks and podcasting. The use of social media has grown exponentially and time spent on social media sites now represents one in five minutes spent online. Concomitant with this online growth, there has been an inverse trajectory in direct face-to-face patient-provider moments, which continue to become scarcer across the spectrum of health care. In contrast to standard forms of engagement and education, social media has advantages to include profound reach, immediate availability, an archived presence and broad accessibility. Our opportunity as health care providers to partner with our patients has never been greater, yet all too often we allow risk averse fears to limit our ability to truly leverage our good content effectively to the online community. This risk averse behavior truly limits our capacity to effectively engage our patients where they are -- online.

## Background

Historically medicine has been viewed as a moral and ethical endeavor with clearly drawn convictions of right and wrong. However, in the last two decades there has been growing unease with a perceived attrition in our duty to patients. This reflects a variety of threats, to include the evolving commodification of medicine, the development and impact of aggressive marketing and the shift from one directional medical interaction to frank patient-provider partnership. These trends, and our responses to them, threaten to jeopardize our ability to partner with our patients effectively in recovery [1]. Nowhere is the perceived opportunity and threat more starkly delineated than in our potential engagement with and silent response to our patients online.

When we discuss medical morality and medical ethics, what we are really referring to is our core belief of what is the right medical action and what is the wrong medical action; in essence, the code by which we practice. The first historic archetype of this from which our current ethics have evolved was likely the Formula Comitis Archiatrorum, written in the 5th century during the reign of the Ostrogothic king Theodoric, preserved by Cassiodorus. Notably, it required that physicians widen and deepen their knowledge and originated our current concept of physician-to-physician engagement and consultation [2].

The six core values codified in current medical ethics include:

• autonomy - the right to refuse or choose treatment (Voluntas aegroti suprema lex);

• beneficence - the need to act in the best interest of the patient (Salus aegroti suprema lex);

• non-maleficence - "first, do no harm" (primum non nocere);

• justice - the burden and the benefit of new treatment must be distributed equally; and

• respect for persons - the patient has the right to be treated with dignity and honesty.

Essentially, our core values encompass our capacity as health care providers to walk with our patients on their journey through illness to recovery. In the current digital era, doing this well may include a seventh precept: that we engage them where they are - online in social media (participes in socialis media).

## Trending Behavior

Two overlapping and powerful trend clusters make this necessary. The first centers on information, reflecting the combined impact of the explosion of medical information and its increasing transparency as we transition to an open access format. The second trend centers on time, reflecting the combined impact of the decreased time providers have in fewer moments of direct patient-provider interaction (partly due to the commodification of medicine[3]), and the rapidly growing time spent online in social platforms by our patients and more recently by providers.

The information overload is striking and profound. PubMed http://www.ncbi.nlm.nih.gov/pubmed/ currently classifies nearly 22 million citations [4], with a new publication being added every minute on average--a rate that has more than doubled over the past 20 years [5]. In my field of practice alone, advanced heart failure and transplant cardiology, 482 guidelines that reference heart failure management are available for review and incorporation in clinical practice in the National Guideline Clearinghouse [6].

Concomitant with this accelerating information overload, our medical information has become more broadly and transparently available, leveling the patient-provider playing field as data increasingly moves to an open access model. Moreover, early access is no longer weighted towards healthcare providers, as late-breaking clinical trials are now often highlighted in publicly available press releases before they appear in peer-reviewed journals, placing additional temporal tension on patient-provider interactions.

Given these two simultaneous information trends, namely vast data overload and increasing information access, the idea that accurate and meaningful information can only flow in one direction, from a provider to a patient, can no longer pass the straight-face test.

The second trend cluster centers on our most precious commodity-time. Face-to-face patient-provider interaction has become scarcer across the spectrum of health care, affecting all providers. Paperwork, particularly documentation, consumes up to one-third of a physician's workday [7]. Physicians-in-training are affected to an even greater degree, with residents spending up to six hours a day in documentation [8], nearly twice as much as twenty years ago[9]. With duty hour restrictions, it would appear that they are at risk of devoting more of their schedule in the future to documentation than to direct patient care.

Moreover, this problem is not unique to physicians; it is frankly pandemic. A recent time-motion analysis of floor nurses demonstrated that less than 20 percent of their shift was spent in direct patient care, with the largest block, 35 percent, spent in documentation [10]. Fundamentally, evolving and competitive demands placed upon health care providers continue to limit opportunities for direct patient-provider communication.

Simultaneously, time spent in social media has grown explosively. As of 2010, the world spends over 110 billion minutes per day on social networks and blogging sites, translating into 22 percent of all time spent online. Time on-line continues to increase exponentially, with the average user spending nearly 6 hours a day on social media sites as of 2010 [11]. Concurrently with more time spent in social platforms, when online, patients and their caregivers are more commonly online searching for medical information, as well as seeking peer-to-peer support. This online health care presence is increasing at a rapid rate; 61 percent of patients are now seeking both support and medical information online [12], and looking for health care information is now the third most popular online activity, after internet search and e-mail [13].

Fundamentally, this represents the opportunity. Much like Alice in Wonderland who shrank when she drank the "DRINK ME" bottle and then bumped her head as she grew after eating the "EAT ME" cake, we face dual challenges as moments of direct patient care that continue to shrink and information for clinical care continues to grow at an astonishing rate. Fortunately, our information is more and more transparent and open sourced, and we know where our patients are. They are online, and they are awaiting our participation.

## The Shape of Partnership

Primarily, our engagement needs meet one of two requirements, either that of content creation or content curation, both of which have profound value to patients and caregivers. Before you begin to engage your patients online, be clear that you have developed and reviewed your organizational social media policy guide. Define the opportunity and operational goals that you intend to address, and remember you represent your organization as well as yourself. Know and review your privacy settings, and ensure that you review them on a regular basis. Learn the rules of the road before driving; our Mayo Clinic Center for Social Media offers the online equivalent of drivers training. Start by observing; spending some time as a lurker can only enhance your experience.

Once online, be real, be professional and be respectful. Most importantly, just like a good marriage, you will be judged more by how you listen than what you say.

## Conclusions

The Cheshire cat is unique in Wonderland. It fears no one, maintaining an outsider status and dispassionate demeanor. It possesses insight into all of Wonderland, and although it seems to speak to Alice in a nonsensical way, in reality the Cheshire cat fully comprehends that it is Alice's behavior that is discordant with the rules of Wonderland, something the cat understands all too well.

Too often we as providers let risk averse fear regarding health care social media guide us to just such a position, remaining aloof and above the opportunity. But while social technologies are wonderful, they do not follow the alien rules of Wonderland. Social media rules are much more in keeping with familiar small-town mores and the ethical obligations of health care providers, but with a technological twist.

We need to engage with our patients as they walk through their journey. The opportunity to do so by participating in social media is profound, extends the reach of content in a scalable fashion, and can be executed without significant cost limitations. We must leverage the content, leverage the conversation, and leverage the good.

We need to be present online, more than the grin of a Cheshire cat.

## Author Information

Twitter Username: @FarrisTimimi

Farris Timimi, MD, is an Assistant Professor of Medicine at the Mayo Clinic College of Medicine, and a Consultant in Cardiovascular Diseases and Internal Medicine at the Mayo Clinic. He serves as Medical Director for the Mayo Clinic Center for Social Media. He serves as the Program Director for the Advanced Heart Failure and Transplant Fellowship Program. In addition, Dr. Timimi is the physician lead for the Division of Cardiovascular Disease One Voice patient-family advisory council.

## Pre-publication history

The pre-publication history for this paper can be accessed here:

http://www.biomedcentral.com/1741-7015/10/83/prepub
